# Sex Differences in Using Spatial and Verbal Abilities Influence Route Learning Performance in a Virtual Environment: A Comparison of 6- to 12-Year Old Boys and Girls

**DOI:** 10.3389/fpsyg.2016.00258

**Published:** 2016-02-25

**Authors:** Edward C. Merrill, Yingying Yang, Beverly Roskos, Sara Steele

**Affiliations:** ^1^Department of Psychology, The University of AlabamaTuscaloosa, AL, USA; ^2^Department of Psychology, Sun Yat-Sen UniversityGuangzhou, China

**Keywords:** sex differences, route learning, development, spatial abilities, verbal memory

## Abstract

Previous studies have reported sex differences in wayfinding performance among adults. Men are typically better at using Euclidean information and survey strategies while women are better at using landmark information and route strategies. However, relatively few studies have examined sex differences in wayfinding in children. This research investigated relationships between route learning performance and two general abilities: spatial ability and verbal memory in 153 boys and girls between 6- to 12-years-old. Children completed a battery of spatial ability tasks (a two-dimension mental rotation task, a paper folding task, a visuo-spatial working memory task, and a Piagetian water level task) and a verbal memory task. In the route learning task, they had to learn a route through a series of hallways presented via computer. Boys had better overall route learning performance than did girls. In fact, the difference between boys and girls was constant across the age range tested. Structural equation modeling of the children’s performance revealed that spatial abilities and verbal memory were significant contributors to route learning performance. However, there were different patterns of correlates for boys and girls. For boys, spatial abilities contributed to route learning while verbal memory did not. In contrast, for girls both spatial abilities and verbal memory contributed to their route learning performance. This difference may reflect the precursor of a strategic difference between boys and girls in wayfinding that is commonly observed in adults.

## Introduction

Wayfinding is commonly defined as an ability to identify one’s current location and successfully navigate to an unseen location in the environment (e.g., Blades, 1997; Montello, 2005). In our everyday environment we often follow the same route from home to school/work and back each day. However, there are times when we may need to negotiate a different route due to some obstruction. In addition, we often need to locate new places in our home environment or travel to unfamiliar destinations. These all involve wayfinding and are integral to efficient daily functioning.

In a seminal paper on the mental representations of large-scale (i.e., real life) environments, Siegel and White (1975) proposed that the acquisition of spatial knowledge of new environments involves three distinct, developmental types of knowledge: landmark knowledge, route knowledge, and configural knowledge. Landmark knowledge refers to knowledge about individual objects in the environment (e.g., the clock tower is tall and has clocks on the back and front). Landmarks are often used as guides for navigating in the environment as well as learning the environment (e.g., “the [destination] is near the clock tower downtown”). Route knowledge reflects knowing a specific route through an environment. It typically includes a sequence of landmarks and the turns necessary to reach a destination (e.g., “Turn right at the clock tower”). Configural knowledge comprises the integration of landmark and route knowledge from multiple experiences into an overall mental representation of the layout of the environment, often referred to as a cognitive map or survey of the environment.

Siegel and White (1975) suggested that the development of first landmark, then route, and then survey knowledge reflected not only the sequence of learning when a person first learns a new environment, but also a developmental progression as a child grows older. That is, young children can remember primarily landmarks, but as they grow older their brains mature enough to remember route and then survey knowledge. The sequential and hierarchical nature of the model has been questioned (e.g., Montello, 1998). However, the relative uniqueness of these three types of spatial knowledge suggests that different cognitive mechanisms may be at work for each. Finally, there are reliable sex differences favoring males at least in terms of memory for and use of survey (configural) knowledge, suggesting that different cognitive mechanisms may be at work between males and females.

In this paper we focus on differential cognitive mechanisms contributing to sex differences in children’s wayfinding, specifically route learning, as they age from 6 to 12 years. We focus first on adults, discussing cognitive predictors of wayfinding and wayfinding differences between men and women. Then we discuss the development of wayfinding in children, followed by predictors and differences between boys and girls.

### Wayfinding in Adults

In a typical route-learning task, a person follows a specified route through an environment that has landmarks located strategically along the route. Sometimes the environment is an actual outdoor or indoor environment, but most often the environment is virtual. There is very little difference in route-finding actions between natural and virtual environments (Kuliga et al., 2015). Further, knowledge learned after active exploration in virtual environments readily transfers to the real world (Richardson et al., 1999; Farrell et al., 2003; Waller et al., 2004; Schinazi et al., 2009; Rodrigues et al., 2010).

To learn the route, most often the person follows verbal directions by an experimenter (e.g., turn right at this corner). After reaching the destination, the participant is taken back to the beginning of the route and s/he is expected to travel the same route without help from the experimenter. During this time, the experimenter notes any errors (e.g., turning left when it was a right turn) and keeps a stopwatch running to measure time to traverse the route. After reaching the destination, the participant is brought to the beginning again, and this continues until the route is followed without error. Hence, the main measure of route learning is the number of trials or the total amount of time needed to learn the route perfectly. Sometimes, after learning the route to criterion, participants are asked to retrace the route from the final destination to the origin; in this case, the dependent measures are the number of errors or amount of time to return to the origin.

Memory for the environment is captured through tests of landmark knowledge and configural knowledge. Landmark knowledge is measured by simply asking the participant for all the landmarks s/he can remember and calculating the proportion of landmarks recalled. Configural knowledge is measured through errors in distance and direction estimations. For example, an experimenter could stop the participant at one of the landmarks in the environment and ask him/her to point in the direction of landmark x (i.e., measuring angular degree of error), and estimate how far the landmark is (i.e., measuring magnitude of estimation error). Configural knowledge is also captured through map drawings made by participants after the task (i.e., the accuracy of the drawings).

#### Cognitive Predictors of Wayfinding in Adults

Several studies have investigated possible cognitive predictors of wayfinding in young adults. Here we focus on three studies that used structural equation modeling (SEM) for more sophisticated analyses. First, Allen et al. (1996) had young adult participants complete object-based spatial ability scales (e.g., cube comparisons), perspective-taking tasks (e.g., recognize whether photo slides were taken from particular locations in a model-sized environment), and route learning tasks with both small scale (e.g., maze on a piece of paper) and large-scale (outdoors) environments. They found that object-based spatial ability indirectly predicted large-scale performance in two different ways. In one case, they predicted performance in the small-scale environment (e.g., trials to learn), which in turn predicted topological knowledge of the large-scale environment (e.g., scene sequencing, route learning). In the other case, object-based spatial abilities predicted perspective-taking performance, which in turn predicted point-to-unseen target performance in the large-scale environment (e.g., distance and angular errors).

Second Kirasic (2000) investigated spatial ability differences in older and younger adults. All participants completed object-based spatial ability tests (e.g., cube comparisons) and spatial ability in a large-scale outdoor environment (e.g., ease of learning the environment, and actual wayfinding behavior in the environment). Age-related differences in large-scale spatial performance (favoring younger adults) were mediated primarily by the small, object-based spatial abilities. Perhaps more important for the current study, overall, object-based spatial ability predicted ease of learning the layout of the environment, which in turn predicted wayfinding behavior.

In the final, most comprehensive study, Hegarty et al. (2006) had participants navigate a route through a virtual environment consisting of four turns and four landmarks. They traversed the route twice. Then, while performing the third trial, participants were stopped at several locations along the way and asked to make distance and direction judgments to two non-visible landmarks (i.e., configural knowledge). At the end of the task, they drew a sketch map of the environment, also a test of configural knowledge. Object-based spatial abilities were measured by tests of mental rotation efficiency, spatial perception of lines within distracting backgrounds, and spatial memory for the locations of objects in a scene. Sense of Direction, a measure of people’s judgments of their own environmental abilities, was assessed using the Santa Barbara Sense-of-Direction Scale (Hegarty et al., 2002). Both object-based spatial abilities and sense of direction significantly predicted a large portion of the variance in configural knowledge (e.g., map drawing, distance and direction estimation). In contrast, verbal abilities as measured by vocabulary and verbal working memory did not predict performance on the tests of configural knowledge. By extension, spatial abilities (but not verbal abilities) would be expected to predict route learning, but this has not been tested extensively using SEM.

#### Sex Differences in Wayfinding in Adults

Another predictor of spatial ability in general and wayfinding in particular is linked to sex differences in the performance of young adults (Linn and Petersen, 1985; Voyer et al., 1995). In fact, many studies have found that men learn spatial environments faster and make fewer errors than do women. For example, Galea and Kimura (1993) had participants learn a route on a novel map and found that men were faster than women at learning the map route. McGuiness and Sparks (1983) had participants draw a map of a familiar territory and found that men were more accurate than women at placing elements in the map. Miller and Santoni (1986) had participants view a map and give directions from memory. They found that men were more likely than women to use Euclidian information, such as cardinal directions and exact distances, and were more accurate in their directions. In their review of the literature, Coluccia and Louse (2004) reported that 61% of wayfinding studies that compared the performance of men and women found better performance in men, with the remaining studies reporting no gender difference. Hence, while differences are not always observed, when they are found it is highly likely that men outperformed women. Coluccia and Louse suggested that the differences in wayfinding between men and women result from males having a greater visual short-term working memory than do women, although they did not test this directly.

A reasonable alternative explanation is that the difference between men and women in wayfinding lies in their use of different strategies rather than their differential abilities or skills (e.g., Lawton, 1994, 2010; Dabbs et al., 1998; Bosco et al., 2004). Men seem to be more likely to use survey/configural strategies (e.g., north, south, east, west) whereas women are more likely to use strategies focusing on landmarks and routes (e.g., left/right). Using survey/configural strategies reflects a more spatially oriented approach in that they involve perceiving, manipulating, and integrating spatial relations between routes. Using landmark/route strategies reflects a more verbal oriented approach in that they commonly involve using verbal labels (i.e., names of landmarks, left/right turns) to create and organize route directions. Consistent with this position, Galea and Kimura (1993) also found that women recalled more landmarks than did men.

Compatible with this strategy-difference hypothesis, Castelli et al. (2008) found relatively strong sex differences favoring adult males in the learning of spatial survey knowledge. This was reflected in accuracy at pointing to the location of landmarks not visible from the current location and placing landmarks on a survey map, which required an integration of information about the spatial layout from different experiences and the efficient use of spatial strategies. In contrast, they did not find significant differences in learning to retrace a route through a virtual environment, which involves the sequential memory of landmarks and turns along a path and can be coded both verbally and non-verbally. Further support for the spatial-verbal divide between men and women comes from EEG studies (e.g., Ramos-Loyo and Sanchez-Loyo, 2011). After wayfinding training in which participants were required to view, explore, and search for specific locations in virtual environments, men showed increased activation of spatial working memory regions in the right hemisphere; whereas women accessed more of their verbal-analytical processing mechanisms in the left hemisphere.

Overall, when there are reliable sex differences in performance on route learning, landmark memory, or configural/survey knowledge, men outperform women. However, the sex differences are larger on tests of configural and/or survey knowledge of the environment. Sex differences in adults’ route learning, as measured by number of trials to learn, has not been investigated as much as in survey learning, as measured by tests of configural knowledge.

### Wayfinding in Children

Developmental studies of wayfinding have necessarily focused on differences between age groups rather than sex differences. Typically, younger children perform worse in wayfinding tasks relative to older children and adults across a variety of methods and approaches (e.g., Cornell et al., 1989; Jansen-Osmann and Fuchs, 2006; Jansen-Osmann et al., 2007a,b). For example, older children are better able to identify and remember useful landmarks than are younger children (e.g., Cornell et al., 1994; Heth et al., 1997; Jansen-Osmann and Wiedenbauer, 2004a). In addition, older children are better than younger children at learning a sequence of landmarks and integrating that sequence into route information (e.g., Jansen-Osmann and Wiedenbauer, 2004a,b). Further, older children are better able to integrate information across routes and use Euclidean information about the common environment than are younger children (e.g., Smith et al., 2013). There is also considerable age-related improvement in the ability to make use of representations of the environment for purposes of navigation (e.g., Blades and Medlicott, 1992).

#### Cognitive Predictors of Wayfinding in Children

There are data suggesting that several basic cognitive abilities correlate with route learning performance in children. Purser et al. (2012) evaluated the relationship between route learning performance of 67 children who were 5- to 11-years-old and several measures of basic cognitive abilities including inhibition, verbal short-term memory, visual short-term memory, verbal long-term memory, and visual long-term memory. They found significant correlations between route learning and all of the basic cognitive abilities. Interestingly, the relationships between route learning and visuo-spatial memory and verbal memory were mediated by performance on the inhibition task. Hence, Purser et al. (2012) concluded that the observed relationships were primarily due to the executive control contributions to each memory task.

These results are consistent with Fenner et al. (2000), who found that an aggregate measure of visuo-spatial ability positively correlated with route learning in children between the ages of 5 and 11 years old. However, Fenner et al. (2000) did not find a significant relationship between route learning and an aggregated measure of verbal ability, which included measures of such things as participants’ knowledge of vowels and word endings, verbal comprehension, general verbal knowledge, and verbal reasoning, as well as verbal short-term memory. It may be that the impact of verbal memory was overshadowed by the inclusion of other measures in the aggregate verbal score.

In another study, Purser et al. (2015) found that measures of attention and long-term memory were associated with route learning for children between 5 and 11 years of age. Hence, while Hegarty et al. (2006) have argued that small-scale spatial abilities may be more highly correlated with survey learning than route learning in young adults, the results of research with children indicate that small-scale abilities may be highly correlated with the route learning performance of children.

The only study to not find a relation between object-based spatial ability and spatial knowledge of an environment was one by Quaiser-Pohl et al. (2004). In their study, 7- to 12-year olds completed three object-based measures of spatial ability (e.g., mental rotation) and also drew a sketch map of their neighborhoods. Using SEM, they found no correlation between the small-scale, object-based measures of spatial ability, and the accuracy of their drawings of their large-scale neighborhoods. However, it is not clear how well the children’s memories for their neighborhood reflect wayfinding ability.

#### Sex Differences in Wayfinding in Children

Only a few studies have reported sex differences in wayfinding activities between boys and girls. In general, results of developmental studies are consistent with those of adult studies. For example, Gibbs and Wilson (1999) compared the wayfinding performance of 51 girls and boys between 5 and 12 years of age. Children were shown a route on a map that included pictures of landmarks and subsequently asked to retrace the route. After correctly retracing the route twice without error, they were asked to remember any landmarks and street names that they could. Boys were able to retrace the route with fewer errors and in less time than did girls. However, girls were able to remember more landmarks (see also Beilstein and Wilson, 2000). These data suggest that boys may be better at wayfinding than girls, and also that boys and girls may use different strategies during route learning. In particular, the finding that girls remembered more landmarks suggests the use of verbal coding during girls’ route learning. Jansen-Osmann and Wiedenbauer (2004a) presented results that support this possibility. They had 2nd grade children, 6th grade children, and young adults learn a route through a virtual environment with landmarks. The participants subsequently retraced the route without landmarks during an initial test phase. Following the first test phase, they relearned the route with landmarks, were shown the route without landmarks, and then attempted to recall the identity and location of landmarks. Boys outperformed girls and men outperformed women when retracing the route without landmarks, indicating a greater reliance on coding of landmarks for females. Interestingly, in the landmark recall task there was an interaction of age and sex such that the young boys recalled more landmarks than the young girls, but women recalled more landmarks than did men. However, in this study there were only 7 boys and 13 girls in the youngest age group.

In addition to the results of wayfinding performance, the results of recall memory in wayfinding tasks are also consistent with girls engaging in verbal coding of the environment more than boys do. For example, Schmitz (1997) had 10- to 12-year-old children explore a real life maze. Written descriptions of the activity produced after several explorations indicated that girls were more likely to include landmark information whereas boys were more likely to include direction information. Similarly, Choi and Silverman (2003) asked 9- to 13-year-old children to first learn a map and then provide written directions from one place to another place on the map. The frequencies of using cardinal directions, relative directions (e.g., left/right), distances, and landmarks were counted. In general, 12- to 13-year-old girls preferred using landmark information over distance information relative to boys of the same age. Spatial perception (as measured by Piaget’s water level task) was positively correlated with boys’ but not girls’ preference to use landmarks.

The study of sex differences in wayfinding performance of children suffers from at least two important weaknesses. First, several of the studies were not specifically designed to evaluate sex differences and hence had limited power to uncover differences that may have been present (i.e., the number of males and females within an age group could be as few as 7). Second, studies that were designed to evaluate sex differences in wayfinding performance of children included a wide range of ages without evaluating developmental changes (e.g., Gibbs and Wilson, 1999; Beilstein and Wilson, 2000) or involved a limited age range across different groups (e.g., Choi and Silverman, 2003). Hence, they were not able to address many questions about the presence of sex differences in wayfinding during the ages when wayfinding skills develop most rapidly.

With the exception of the correlation between visual perception ability and boys’ but not girls’ preference to use landmarks as reported by Choi and Silverman (2003), there is no data available on cognitive predictors of sex differences in wayfinding of children. However, based on the accumulated evidence from adults and children, it may be predicted that (a) both object-based spatial ability and route learning will increase as age increases; (b) verbal and visual measures of working memory will increase as age increases; (c) there will be sex differences in object-based spatial ability and route learning; and (d) because men and women tend to use different wayfinding strategies, it may be the case that boys’ and girls’ route learning will depend on different strategies. Specifically, boys’ route learning may be predicted more strongly by visual (working memory) and/or spatial (object-based) measures compared to girls. Also, girls’ route learning may be predicted equally by verbal (working memory; vocabulary) and visual-spatial measures, whereas boys’ route learning would not.

### Current Study

In this research, the primary goal is to investigate cognitive contributors to sex differences in wayfinding of children 6-to 12-years-old. To accomplish this goal, we investigated the relationship between route learning performance and small-scale spatial abilities and verbal memory in girls and boys. In this study, spatial abilities were measured by spatial working memory task, a version of Piaget’s Water Level task to measure spatial perception and a mental paper folding task to measure spatial visualization. In the paper folding task, participants have to mentally fold a piece of paper, imagine a hole punched through the folded paper, and determine what the paper would like when unfolded. We did not expect girls and boys to differ on spatial abilities, because such differences do not typically emerge until children are at least 12 years old (Voyer et al., 1995). Verbal memory was measured by a word list learning task. To measure route learning, we had participants learn a path through series of hallways presented via a virtual environment. Based on previous research (e.g., Gibbs and Wilson, 1999; Beilstein and Wilson, 2000; Jansen-Osmann and Wiedenbauer, 2004a) we expected that boys would outperform girls in the route learning task. To the extent that differences in route learning are related to sex differences in how girls and boys learn the route, we would also expect a different pattern of associations between small-scale spatial abilities and verbal memory to be apparent for boys and girls. More specifically, we expected the performance of boys to be more closely associated with performance on the spatial abilities measures and the performance of girls to be more closely associated with verbal memory.

We chose a virtual environment to assess route learning in this study. This option was selected for several reasons. First, there are clearly safety and anxiety concerns associated with walking young children around an unfamiliar environment. Safety concerns would limit the willingness of parents to grant permission. Anxiety on the part of the young children might have influenced the ability to assess the contributions of cognitive variables. Second, a real environment would require the presence of the experimenter in close proximity and affect the degree to which participants would view their activity as independent. Third, the use of a virtual environment helps create a well-controlled environment, limiting the number of extraneous variables that can influence performance. Small-scale spatial abilities have generally correlated higher with wayfinding acquired through virtual environments or video experience than in real life environment (Hegarty et al., 2006), perhaps because virtual environments provide greater control over extraneous variables. It is also the case that in spite of concerns about using virtual environments to study wayfinding (e.g., Ruddle et al., 1997), knowledge learned after active exploration in virtual environments readily transfers to the real world environments (Richardson et al., 1999; Farrell et al., 2003; Waller et al., 2004; Schinazi et al., 2009; Rodrigues et al., 2010).

## Materials and Methods

### Participants

All recruitment and testing procedures followed the guidelines of the university Institutional Review Board. We recruited and tested 154 participants aged 6- to 12-years-old (145 Caucasians, 7 African American, and 2 Asians) from local afterschool programs, private schools, homeschools, churches, and via local ads. Approximately 80% of the children were from middle class families. Due to a technical failure, one child’s data were not recorded. In the final sample, there were 80 boys (mean age: 9 years and 9 months, *SD* = 1.59 years) and 73 girls (mean age: 9 years and 6 months, *SD* = 1.54 years). A *t*-test indicated no significant difference between the mean ages of the boys and girls [*t*(151) = -0.72, *p* = 0.47]. Each child participant received a $10 gift card for completing the study.

### Materials and Procedures

Participants were tested individually in a quiet location in their schools, afterschool programs, homes, or our lab at the university, whichever was convenient for the parents. Testing time ranged from 40 to 75 min. The order of the tests was counterbalanced across participants. Each measure is described below. Cronbach’s alphas are reported for the current data.

#### Word Learning Test

This task measures verbal learning and memory over repeated presentations. It was patterned after the modified NEPSY (“A Developmental Neuropsychological Assessment”; Korkman et al., 1998) list learning task used by Pennington et al. (2003). The participants were read a list of 15 words by a native English speaker. After the presentation of the entire list, participants were asked to recall as many words as possible. The list, followed by immediate recall, was repeated up to five times. Participants who recalled the entire list prior to the fifth repetition were not required to continue and were given credit for recalling all words for each remaining repetition. The total score across five trials was calculated for each participant as a measure of verbal memory (Cronbach’s α = 0.88). No participant performed at ceiling (i.e., recalling the entire list on the first attempt).

#### Mental Rotation

This task was a two-dimensional mental rotation task (Cooper and Shepard, 1973; Hinnell and Virji-Babul, 2004). More specifically, the letter F or its mirror image (along the vertical axis) was rotated different degrees (0, 45, 90, 135, 180, 245, 270, and 315) and presented on a computer screen. The participants’ task was to decide whether it was a typical or mirror image F. Responses were made by pressing the corresponding key on the keyboard. Before the test started, each child was provided four practice trials that included two typical and two mirror image Fs rotated 0 or 45°. The test did not start until the child clearly demonstrated his/her understanding of the task. All children successfully completed the practice trials. During the test, there were 10 blocks of trials including eight typical and eight mirror Fs rotated various degrees each for a total of 160 trials. A break was provided after each block. Seven of the children made over 60 errors (>38%), and their data on this task were discarded and coded as missing data. For the rest of the participants, the average error rate was 8.1%. For each child, we calculated the mental rotation slope by regressing reaction times on absolute angle of rotation (0, 45, 90, 135, and 180). Rotation slopes were used in the final data analysis (Cronbach’s α = 0.65) because they are generally considered a good measure of mental rotation efficiency when error rates are reasonably low (Cooper and Shepard, 1973). The average R squared of the linear regression was 0.26 (*SD* = 0.13), and significantly greater than 0, *p* < 0.001.

#### Visuo-Spatial Working Memory

This task was patterned after Simmering (2012). Participants were shown a series of displays presented in pairs on a computer screen. The first display of each pair (the sample display) was presented for 250 ms, followed by a blank screen for 750 ms. The second display in each pair (the test display) was presented until a response was made. Each display included 3–8 colored squares presented in random locations. The sample and test displays in each pair included the same number of squares in the same locations. The test display was either identical to the sample display or one of the squares was in a different color. The participants’ task was to indicate whether the test display was the same as or different from the sample display using the keyboard. There were eight test pairs of each display size for a total of 48 pairs/trials. We recorded the total number of correct responses for each participant. More correct responses indicated higher visuo-spatial working memory capacity (Cronbach’s α = 0.74).

#### Water Level Task

In this Piagetian paper-pencil task (Piaget and Inhelder, 1956), participants viewed 12 different containers whose degrees of deviations from upright ranged from 25 to 45°. Participants drew a line to represent a half-filled level of water inside the container. Two different raters independently measured the degree of deviation from horizontal of the water lines that participants had drawn. The inter-rater reliability was very high (Cronbach’s α = 0.99). The average degree of deviation calculated by the two raters was used as the participant’s score for each picture. Based on their average performance across 12 pictures, the participants were classified as exhibiting high, medium, or low spatial perception (Lohaus et al., 1996). The high group included participants who exhibited average deviations of less than 10° (14 participants). They appeared to be able to correctly use the ground as a reference of frame, although their depiction of horizontal lines was not perfect. The medium group of participants exhibited average deviations were between 10 and 34 degrees (20 participants). These participants may understand that using the container as the frame of reference was incorrect but did not understand the use of ground level as the appropriate reference. The low spatial perception group was the largest group of participants. They exhibited average deviations over 35° (119 participants) and appeared to be using the containers’ bottom as the frame of reference.

#### Route Learning Task

**FIGURE 1:**
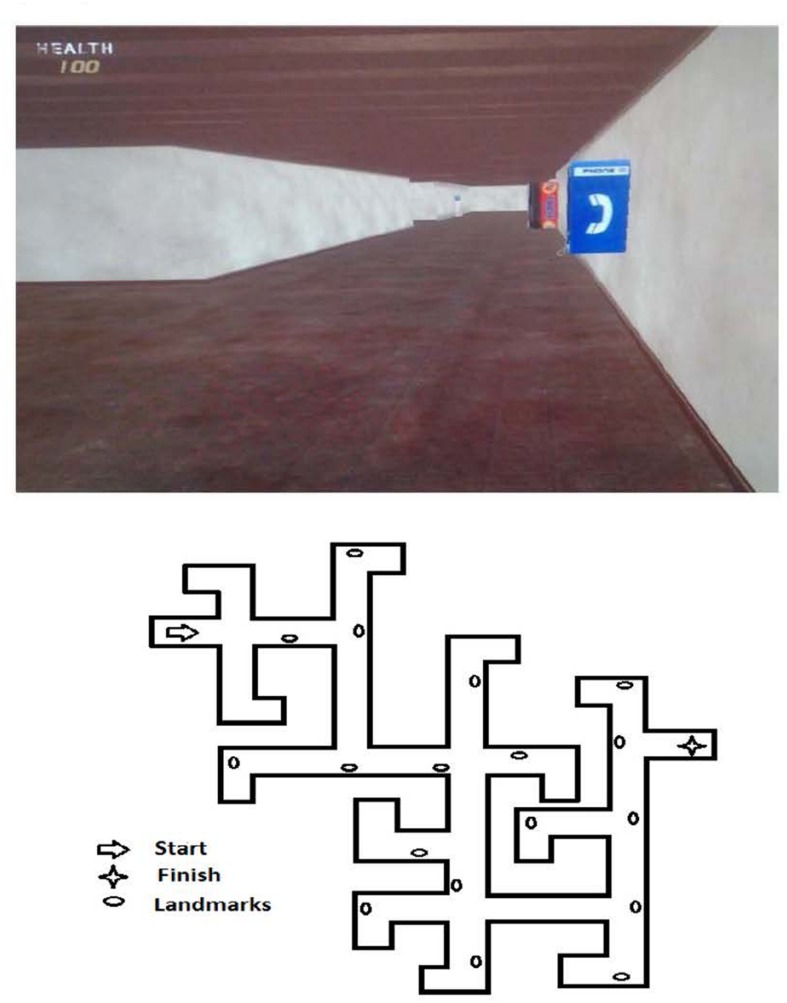
**A screenshot of one segment of the route (top) and birds eye view of entire route (bottom)**.

This task was identical to the one used by Davis et al. (2014; see **Figure 1**). The route was programmed using the FPS CREATOR virtual environmental tool (FPS refers to First Person Shooter, although no combat/shooter props were used in the study, http://fps-creator.en.softonic.com/). This virtual environment consisted of a set of hallways with eight choice points along the way. Half of the choice points included two turning options and the other half included three turning options. The correct route to the destination included going straight at two choice points, turning right at three choice points, and turning left at three choice points. Sixteen unique landmarks were placed against the walls along the route, with half of the objects adjacent to choice points and the other half at non-choice points. All of the landmarks were appropriate for an indoor hallway (e.g., blue phone, ice machine, blue dolly, gumball machine, water fountain, drink machine, fan, and security camera). Participants navigated the route using both the computer mouse (for turning) and the “w” key on the keyboard (for moving forward). Before they started the actual task, the experimenter taught children how to use the mouse and keyboard as needed in an open room environment and made sure that they had no problem controlling them. Then the participants were told that they were going to be shown a path through a set of hallways to find a scientist (the final destination). They were advised to pay close attention because they were going to navigate the path themselves later. The experimenter traveled the path showing the participant the correct route to the destination. Next the program was restarted and the child was asked to find the scientist.

An error was recorded if participants made a wrong turn and entered an incorrect hallway, or if they returned to where they already passed. Simply looking down an incorrect hallway was not considered an error. If a participant returned to a previously passed hallway, he or she was stopped by the experimenter, told that they had been that way already, and asked to choose a different path. This approach was necessary to avoid frustration. Participants were given up to 10 trials to reach the criteria of navigating the route without any error. The total number of trials to reach criterion was recorded by the experimenter. If they still made errors on the 10th attempt, the number of trials was recorded as 11. All but four children received a score of 10 or better. After reaching the criteria, participants were asked to reverse the route and go back to where they started once. Errors on the reverse trial were recorded. Finally, participants were asked to recall all the objects they saw in the maze without seeing the route again. Number of errors and number of trials to learn the route on forward attempts, number of errors on the reverse trial, and number of landmarks recalled were measures of route learning performance.

## Results

### Data Analysis Plan

For convenience, throughout the results section the generic term wayfinding was used to refer to the aggregated measure of route learning performance. The data analysis was conducted in several steps. First, descriptive statistics were calculated and evaluated in preparation for the SEM analyses. SEM was subsequently used to develop a model relating Sex, Age, Spatial Abilities, and Verbal Memory to Wayfinding Performance. Finally, a multi-group analysis was conducted to determine whether the structural models were different for boys and girls.

### Descriptive Statistics

Descriptive Statistics for each measure and the correlation matrix are presented in **Table 1**. These correlations were exploratory. Hence, we corrected for increased Type I error by setting alpha at *p* < 0.01. We found that age significantly correlated with all ability measures, with increases in age being associated with better performance on all measures (paper folding, visuo-spatial working memory, mental rotation, water level task, word learning, and the four measures of wayfinding). Sex significantly correlated with three of the four measures of wayfinding (errors reverse, total trials, and errors forward), but not with measures of spatial ability or verbal memory. Boys had higher performance levels than girls on all wayfinding variables: fewer trials to reach learning criteria, fewer errors finding the destination, fewer errors reversing the route, and more landmarks recalled.

**Table 1:** Raw correlations, means, and standard deviations of all the participants.

	1	2	3	4	5	6	7	8	9	10	11
(1) Age											
(2) Sex	-0.07										
(3) Water level	-0.32ˆ**	0.19									
(4) Mental rotation (slope)	-0.23ˆ**	0.06	0.15								
(5) Spatial working memory	0.54ˆ**	-0.07	-0.28ˆ**	-0.18							
(6) Paper folding	0.34ˆ**	0.04	-0.32ˆ**	-0.24ˆ**	0.20						
(7) Word learning	0.32ˆ**	0.02	-0.09	-0.13	0.25ˆ**	0.19					
(8) Reverse errors	-0.18	0.26ˆ**	0.12	0.09	-0.24ˆ**	-0.07	-0.15				
(9) Total trials	-0.38ˆ**	0.29ˆ**	0.29ˆ**	0.21	-0.37ˆ**	-0.25ˆ**	-0.24ˆ**	0.21ˆ**			
(10) Total errors	-0.45ˆ**	0.29ˆ**	0.27ˆ**	0.17	-0.37ˆ**	-0.25ˆ**	-0.26ˆ**	0.21ˆ**	0.92ˆ**		
(11) Landmarks recall	0.20	-0.16	-0.20	-0.08	0.09	0.12	0.26ˆ**	-0.27ˆ**	-0.24ˆ**	-0.26ˆ**	
Mean	9.65	1.48	2.69	300.55	34.26	6.00	40.09	1.54	3.86	8.22	5.30
SD	1.57	0.50	0.63	217.23	6.15	2.20	9.87	1.99	2.53	9.55	1.84

*^∗∗^*p* < 0.01*.

**FIGURE 2:**
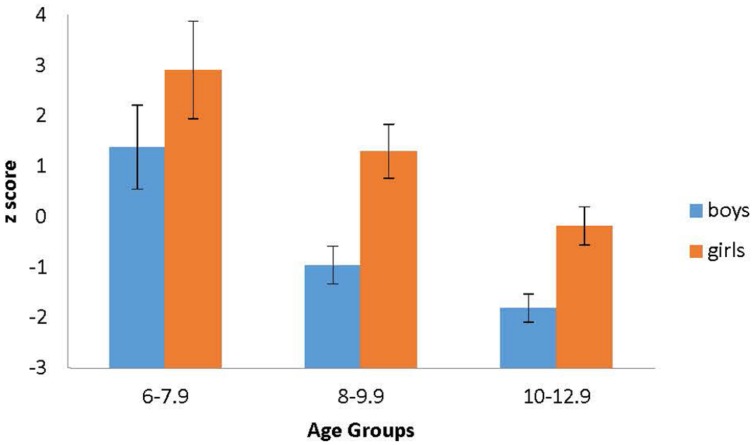
**Aggregated wayfinding *z* scores by sex and age**. Larger aggregated *z* scores indicated more wayfinding errors and worse overall wayfinding performance. Error Bars: ±1 SE.

To evaluate whether the wayfinding difference between boys and girls was related to participant age, we divided the sample into three age groups; 6 – 7.9 years (12 boys and 17 girls), 8 – 9.9 years (35 boys and 25 girls), and 10 – 12.9 years (33 boys and 31 girls). The general characteristics of each age group are reported in **Table 2**. The oldest group included a wider range because relatively few participants were older than 12.0 years of age. We then calculated an aggregate *z* score for each participant using the four wayfinding measures. More specifically, for each measure, we obtained a *z* score based on statistics of all the participants. For three of the measures, larger *z* scores reflected more errors and hence poorer performance (i.e., errors reverse, total trials, and errors forward), whereas for landmarks recalled a larger score represented better performance (more landmarks recalled). We multiplied the *z* scores for landmarks recalled by -1 (reflecting landmarks not recalled) generating scores in the same direction as the other measures. Then we added the *z* scores of total errors, total trials, errors reversed, and transformed landmarks recalled. The larger aggregate score thus reflected a greater number of errors. These data are presented in **Figure 2**. A 3 (group: 6–7.9; 8–9.9; 10–12.9) × 2 (sex: boys vs. girls) ANOVA on the aggregate *z* score was conducted. The main effect of age group was significant, *F*(2,147) = 15.87, *p* < 0.001, $\eta_{p}^{2}$ = 0.18, with the oldest group (*M* = -1.0, *SE* = 0.31) demonstrating better wayfinding performance than the middle group (*M* = 0.17, *SE* = 0.32), who in turn performed better than the youngest group (*M* = 2.15, *SE* = 0.47). The main effect of sex was significant as well, *F*(1,147) = 17.53, *p* < 0.001, $\eta_{p}^{2}$ = 0.11, with boys (*M* = 0.46, *SE* = 0.31) demonstrating better wayfinding performance than girls (*M* = 1.35, *SE* = 0.30). The interaction was not significant, *F*(2,147) = 0.32, *p* = 0.73. Comparing the performance of girls and boys at each age level, using Bonferroni correction, revealed a significant difference between sexes in the two older groups (both *p*’s < 0.05) and a marginally significant difference in the youngest group (*p* = 0.10) for which there was less power. Taken together, these results indicate that boys performed better than girls in our route learning task and that the magnitude of the difference did not vary across the age range tested.

**Table 2:** Means and standard deviations (in years/months) for age subgroups compared in ANOVA.

Age group	*N*	Mean	Standard Deviation
6 years – 7 years 11 months	29	7 years 5 months	5 months
8 years – 9 years 11 months	60	9 years 1 month	6 months
10 years – 12 years 11 months	64	11 years 2 months	9 months

### Modeling the Relations between Age, Sex, Spatial Abilities, Verbal Memory, and Route Learning

We conducted a SEM analysis using Mplus 7.2. Three latent variables were constructed. The first latent variable was wayfinding as indicated by the four measures in the route learning task: total number of trials forward, number of errors forward, number of errors backward and landmarks recalled. Again, the measure of landmark was transformed to reflect landmarks not recalled. Hence, all four indicators of wayfinding were in the same direction with larger scores indicating more errors. Thus, higher scores in the wayfinding construct would indicate poorer performance in wayfinding. Because trials to criteria and forward errors were correlated greater than 0.90, they were allowed to correlate in the model. The second latent variable was spatial ability. It was indicated by the four spatial tasks: paper folding, water level, spatial working memory, and mental rotation. The third latent variable was verbal memory as indicated by word learning^1^. Higher scores indicated better spatial ability and verbal memory.

**FIGURE 3:**
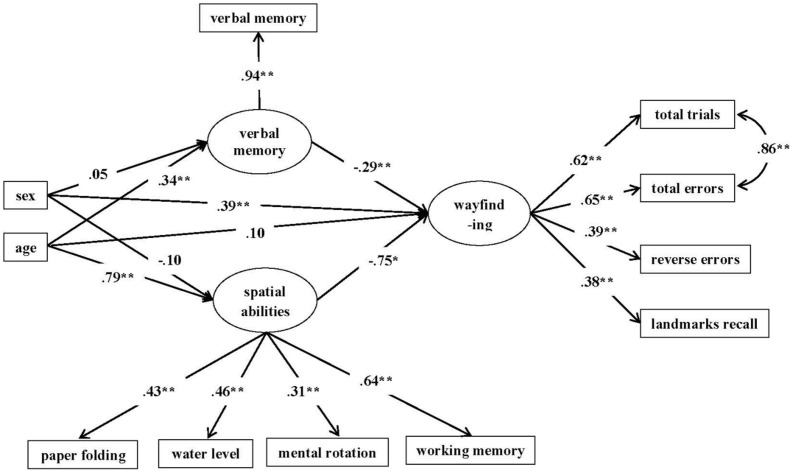
**Full model diagram**. The figure reports standardized parameter estimates. The parameter estimate standardized using only the variances of wayfinding (Muthén and Muthén, 2012) from sex to wayfinding is 0.11. ^∗∗^*p* < 0.01, ^∗^*p* <0.05.

**FIGURE 4:**
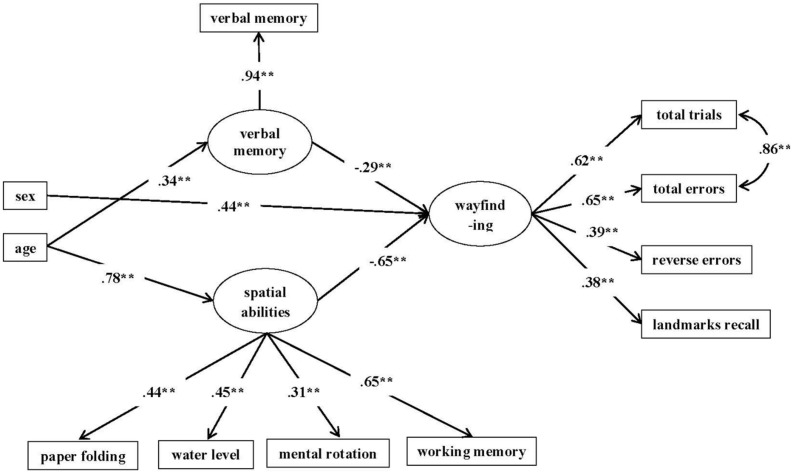
**Model I diagram**. The figure reports standardized parameter estimate, The parameter standardizing the variable of sex is 0.12. ^∗∗^*p* < 0.01.

We employed MLR estimator (maximum likelihood estimation with robust standard errors) rather than ML estimator (maximum likelihood) because it is more robust to violations of multi-variate normality. The Satorra- Bentler-scaled chi-square statistic (S-B χ^2^) was used for comparing fit statistics of the different models. Given that the χ^2^ indicates the deviance of the data-input variance covariance structure from the model-implied variance covariance structure, significant SB χ^2^ indicates the misfit of the data to the hypothesized model. Since χ^2^ statistic is a function of sample size, it is more likely to be significant with a large sample size (Kline, 2011). Therefore, we also employed alternative fit indices, which are, CFI, RMSEA, and SRMR. We employed the standard of CFI values over 0.90 and RMSEA less than 0.08 as indications of acceptable fit (Kline, 2011; Little, 2013). A SRMR value of less than 0.08 is generally considered a good fit (Hu and Bentler, 1999). The full structural-equation model is presented in **Figure 3**. In the model, age and sex were entered as predictors of all three latent variables, with sex coded as 1 for boys and 2 for girls. A positive path coefficient would indicate that girls obtained higher scores and hence performed more poorly than did boys. Spatial ability and verbal memory were also entered as predictors of wayfinding. It was a multiple-indicator-multiple-cause (MIMIC) model in SEM. The results suggested that overall we obtained a good fit (see model fit statistics in **Table 3**). The standardized path coefficients are included in **Figure 3**. As can be seen in the Figure, age significantly predicted both verbal memory and spatial ability. However, age was not a significant predictor of wayfinding. Sex was a significant predictor of wayfinding, yet it did not predict spatial ability or verbal memory. In addition, both spatial ability and verbal memory accounted for a significant portion of variance in wayfinding. However, three paths in the full model were not significant. After dropping these paths, we achieved a more parsimonious model (Model I, see **Table 3** and **Figure 4**), which also did not differ from the full model, S-B χ^2^ (df = 3) = 0.544, *p* > 0.05. Therefore, Model I was chosen as the final model.

**Table 3:** Model fit statistics for full model and Model I.

Model	χ^2^	df	*p*	Scaling error for MLR	RMSEA	90 percent CI of RMSEA	CFI	SRMR
Full model	49.84	38	0.0946	0.9150	0.045	0.000 0.077	0.976	0.048
Model I final model	51.34	41	0.1293	0.9192	0.041	0.000 0.072	0.979	0.052

### Multi-Group Analysis

We conducted a multi-group analysis to determine whether the relationships between verbal memory and wayfinding and between spatial ability and wayfinding identified in the final model were different for boys and girls. **Table 4** presents the correlation matrix of descriptive statistics for boys and girls separately.

**Table 4:** Correlations, means and standard deviations by sex (males: below the diagonal; females: above the diagonal).

	1	2	3	4	5	6	7	8	9	10
(1) Age	1	-0.29	-0.10	0.57ˆ**	0.32ˆ**	0.41ˆ**	-0.26	-0.33ˆ**	-0.45ˆ**	0.16
(2) Water level	-0.34ˆ**	1	0.17	-0.28	-0.23	-0.26	0.14	0.29	0.25	-0.15
(3) Mental rotation (slope)	-0.30ˆ**	0.12	1	-0.17	-0.26	0.07	-0.03	0.22	0.18	-0.03
(4) Working memory	0.51ˆ**	-0.26	-0.18	1	0.22	0.34ˆ**	-0.30ˆ*	-0.34ˆ**	-0.35ˆ**	0.10
(5) Paper folding	0.36ˆ**	-0.42ˆ**	-0.22	0.19	1	0.17	-0.14	-0.20	-0.22	0.11
(6) Word learning	0.23	0.03	-0.28	0.17	0.21	1	-0.12	-0.40ˆ**	-0.43ˆ**	0.30ˆ**
(7) Errors when reverse (RL)	-0.07	0.03	0.19	-0.16	-0.00	-0.21	1	0.14	0.18	-0.44ˆ**
(8) Total trials (RL)	-0.45ˆ**	0.24	0.18	-0.41ˆ**	-0.37ˆ**	-0.09	0.15	1	0.90ˆ**	-0.22
(9) Total errors (RL)	-0.47ˆ**	0.22	0.16	-0.40ˆ**	-0.34ˆ**	-0.08	0.09	0.93ˆ**	1	-0.24
(10) Landmark recall (RL)	0.21	-0.18	-0.10	0.05	0.15	0.25	-0.06	-0.20	-0.22	1
Male mean	9.75	2.58	289.03	34.68	5.92	39.91	1.03	3.16	5.60	5.58
Male SD	1.59	0.71	238.93	6.44	2.16	9.67	1.65	2.04	7.56	1.98
Female mean	9.54	2.81	313.73	33.78	6.08	40.29	2.08	4.63	11.08	4.99
Female SD	1.54	0.52	190.24	5.82	2.27	10.14	2.19	2.80	10.66	1.64

*RL, route learning. ^∗∗^*p* < 0.01*.

To compare the path coefficients between boys and girls, we first established measurement invariance (Milfont and Fischer, 2010; see also Meredith, 1993; Muthén and Muthén, 2009) to ensure that the indicators of the latent variables measured the same psychological construct for the two groups. Only indicators of the latent variables and the latent variables themselves were included in the measurement model. The model fit indices are in **Table 5**. We first achieved the configural model. The parameter between reverse errors and landmarks recall was freed in females according to the modification indices. We also achieved the weak model (i.e., factor loading invariance) and strong model (i.e., factor loading and intercept invariance). There was no difference between the configural model and weak model, S-B χ^2^ (df = 6) = 1.41, ns, and the latter did not differ from the strong model, S-B χ^2^ (df = 6) = 12.12, ns. Hence, the results supported the assumption of invariance in the factor loadings and intercepts between boys and girls.

**Table 5:** Model fit statistics testing measurement invariance models.

Model	χ^2^	df	*p*	MLR scaling error	RMSEA	90 CI RMSEA	CFI	SRMR
Configural model	62.397	49	0.095	0.849	0.060	0.000 0.100	0.969	0.068
Weak model	61.706	55	0.249	0.885	0.040	0.000 0.085	0.984	0.071
Strong model	74.518	61	0.115	0.898	0.054	0.000 0.092	0.968	0.083

After the measurement invariance was established, we compared factor variances and covariances across two sexes (see **Table 6** for model fit statistics). Compared with the measurement model, the structural model here included the paths from age to spatial abilities, from age to verbal memory, from verbal memory to wayfinding, and from spatial abilities to wayfinding, as well as the residual covariance between total trials and total errors. In Model A, the variances of three latent variables between the two sexes were held equal and the structural paths were free to vary. This result suggested a good model fit for Model A. In addition, there was no difference between Model A and the strong measurement model, S-B χ^2^ (df = 20) = 15.26, ns, indicating that the variances of the three latent variables were not different for boys and girls.

**Table 6:** Model fit statistics for testing structural invariance.

Model	χ^2^	df	*p*	MLR scaling error	RMSEA	90 CI RMSEA	CFI	SRMR
**Model A** equal variances of three latent variables; free all covariance paths	89.486	81	0.243	0.907	0.037	0.000	0.983	0.082


						0.076		
**Model B** Equal path verbal memory to wayfinding	94.21	82	0.168	0.909	0.044	0.000	0.976	0.088


						0.080		
**Model C** Equal path spatial abilities to wayfinding	101.432	82	0.072	0.904	0.056	0.000	0.962	0.093


						0.089		
**Model D** Equal path verbal memory to age	90.032	82	0.255	0.036	0.036	0.000	0.984	0.082


						0.075		
**Model E** Equal path spatial abilities to age	90.740	82	0.239	0.909	0.037	0.000	0.983	0.082


						0.076		
**Model F** Equal three latent variable means across groups	116.126	82	0.008	0.904	0.074	0.039	0.933	0.104


						0.103		
**Model G** Equal latent means of spatial abilities and verbal memory, free wayfinding	93.651	81	0.159	0.910	0.045	0.000	0.975	0.083


						0.081		

**FIGURE 5:**
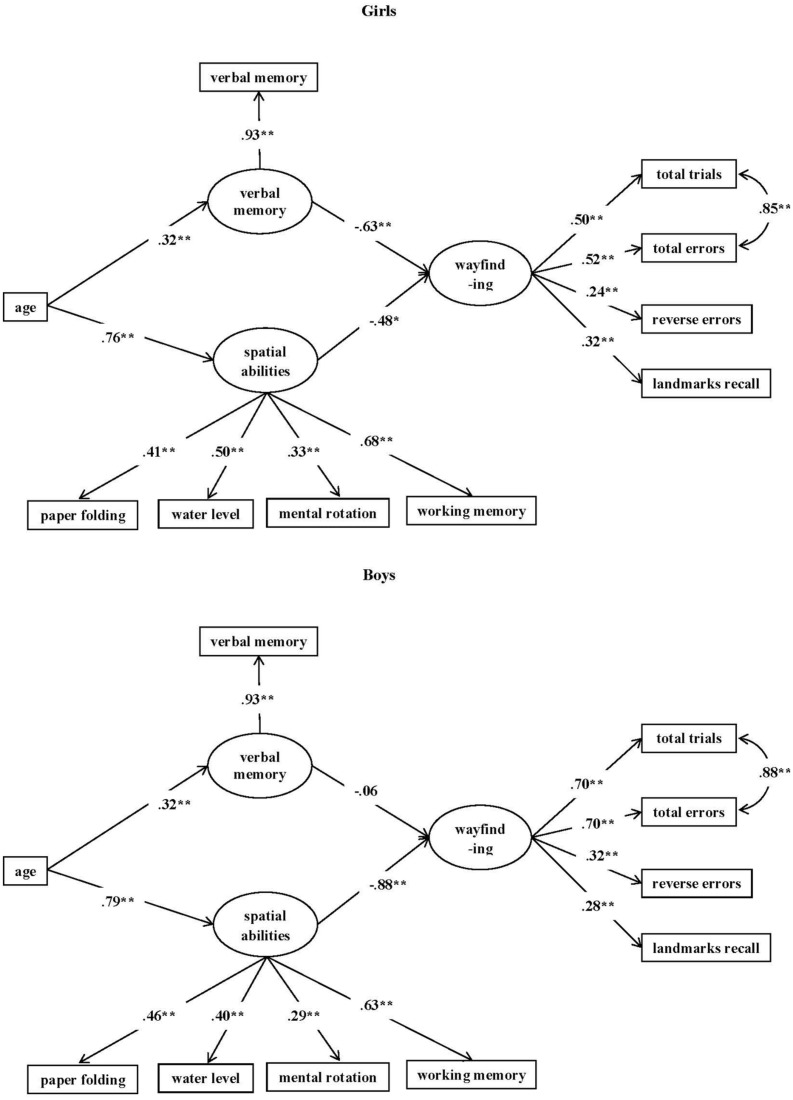
**Model A applied to girls (top) and boys (bottom) separately**. The figure reports standardized parameter estimates. All paths are significant except the path from verbal memory to wayfinding for boys. Although reverse errors and landmark recall was correlated for girls, it was not shown in this diagram. The standardized parameter estimate for reverse errors and landmark recall was 0.39, *p* < 0.005, ^∗∗^*p* < 0.01.

Next we evaluated Model A (see **Figure 5**) for girls and boys separately. For girls both the paths from spatial abilities to wayfinding (β = -0.482, 95% CI using bootstrapping: -0.880 to -0.090) and from verbal memory to wayfinding (β = -0.634, 95% CI: -0.986 to -0.282) were significant. We then compared the path coefficients for girls from spatial abilities to wayfinding and from verbal memory to wayfinding by constraining the two paths to be equal (UCLA Statistical Consulting Group, 2014). The Wald’s test (df = 1) = 0.121, *p* = 0.728 indicated no significant difference between the two path coefficients. Hence, for girls spatial abilities and verbal memory appeared to predict wayfinding to the same degree.

For boys, only the path from spatial abilities to wayfinding was significant (β = -0.883, 95% CI: -1.104 to -0.664); the path from verbal memory to wayfinding was not significant (β = -0.058, 95% CI: -0.314 to 0.202). The two path coefficients were also significantly different from each other, with the path coefficient from spatial abilities to wayfinding being greater in absolute value than that from verbal memory to wayfinding, Wald’s test (df = 1) = 12.436, *p* < 0.001. Hence, it suggested a strong association between spatial abilities and wayfinding and an almost complete dissociation of verbal memory and wayfinding for boys, with spatial abilities contributing significantly more to wayfinding performance than did verbal memory.

We subsequently compared the two sexes directly. We first compared the path from verbal memory to wayfinding across boys and girls. This was done by testing the invariance of factor covariances. In Model B, the structural path between verbal memory to wayfinding and the latent variable variances were held equal across two groups. Model B suggested a good model fit (see **Table 6** for model fit statistics). However, there was a significant difference between Model B and Model A, S-B χ^2^ (df = 1) = 4.40, *p* < 0.05. The path coefficient between verbal memory and wayfinding was significantly larger for girls than for boys. Hence, verbal memory made a larger contribution to wayfinding for girls than it did for boys.

Next, in Model C, we tested the path between spatial abilities and wayfinding for boys and girls in a similar manner as in Model B. Although Model C suggested an acceptable model fit, it was a poorer fit than Model A, S-B χ^2^ (df = 1) = 17.55, *p* < 0.001. The path coefficient from spatial abilities to wayfinding was greater for boys than for girls. Hence, the relationship between spatial abilities and wayfinding was significantly stronger for boys than it was for girls.

In Models D and E, we constrained the paths from age to spatial abilities and from age to verbal memory to be equal across two groups, respectively. Neither was significantly different from Model A, S-B χ^2^ (df = 1) = 1.10 and S-B χ^2^ (df = 1) = 1.23, ns, indicating that the paths from age to verbal memory and age to spatial abilities were not significantly different for boys and girls. Next, we compared latent means between two sexes. In Model F, we equated the means of all three latent variables across two groups (see **Table 6** for model fit statistics). Model F differed significantly from the saturated model (where variances and covarainces are fully explained), *p* = 0.008, and hence was not a good fit for the data. Modification indices suggested that the latent means of wayfinding between two groups should be allowed to vary. Model G incorporated the modification indices by freeing latent means of wayfinding between two groups. Comparing Model G to Model F also indicated that Model G provided a better model fit than Model F, S-B χ^2^ (df = 1) = 52.22, *p* < 0.001. Therefore, the latent means of wayfinding were significantly different across the two groups with boys exhibiting better wayfinding performance than girls. Hence, even though girls and boys exhibited similar levels of performance in both spatial abilities and verbal memory, boys performed significantly better in wayfinding.

## Discussion

In this research, we evaluated the relationships between route learning performance and age, sex, several small-scale spatial abilities and verbal memory using a structural equation modeling approach. We specifically focused on the emergence of route learning differences between boys and girls between 6- to 12-years-old. Our results indicated that both small-scale spatial abilities and verbal memory accounted for a significant portion of the variance in route learning. Of major importance, we found that sex also accounted for a significant portion of the variance in route learning differences above and beyond those predicted by small-scale spatial abilities and verbal memory. Multiple group analysis indicated a different pattern of predictors for boys and girls. More specifically, for boys psychometric spatial abilities significantly contributed to route learning performance while verbal memory did not. For girls, both spatial abilities and verbal memory contributed to route learning performance. We also observed that the correlation between spatial ability and route learning was stronger in boys than in girls; whereas, the correlation between verbal memory and route learning was stronger in girls than in boys. Finally, boys had better overall route learning performance than did girls. Interestingly, there was no evidence that the sex difference in wayfinding varied across the age range tested, indicating that the basis for the observed sex difference was evident at 6 years of age. These results are discussed below.

### Relationship between Age and Route Learning

Age was significantly correlated with our wayfinding indices. However, it did not significantly contribute to wayfinding in the SEM model. When spatial ability and verbal memory were entered into the model, the effect of age was eliminated. Hence, route learning improved with age but not simply because children got older. Rather, it was the fact that spatial ability and verbal memory consistently improved with age. This result is consistent with observations in several domains indicating that changes in more basic abilities with age mediate changes in the expression of general cognitive skills as children get older (e.g., Fry and Hale, 1996; Raghubar et al., 2010). In addition, the failure to observe an independent effect of age suggests that variables other than those measured by our verbal memory and spatial abilities tasks that systematically vary with age did not make a reliable contribution to our participants’ route learning performance.

### Relationship between Small-Scale Spatial Abilities and Wayfinding

Our results suggest that several small-scale spatial abilities (spatial perception, spatial visualization, and visuo-spatial working memory) may be related to route learning for children. While these results are in contrast with studies of adult route learning (e.g., Hegarty et al., 2006), they are consistent with several recent studies of wayfinding performance in children. As discussed in the Section “Introduction,” Purser et al. (2012) reported correlations between specific small-scale spatial/verbal abilities (i.e., spatial memory and verbal memory) and wayfinding competence in children. In addition, Fenner et al. (2000) found that children 5-to 6-years-old with higher visual-spatial composite scores (measured by un-speeded mental rotation, paper folding, and spatial memory) had better real life-route learning performance compared with those with lower visual-spatial abilities. These results indicate that basic spatial abilities may help to explain individual differences in the route-learning performance of children to a greater degree than is typically seen for young adults (e.g., Wolbers and Hegarty, 2010). This is not to say that small-scale spatial abilities do not contribute to route learning for adults. Indeed, adult performance can be compromised when, for example, visuo-spatial working memory is otherwise occupied in a secondary task (e.g., Garden et al., 2002; Meilinger et al., 2008). However, it may be that these abilities are no longer sufficient to account for variations seen in wayfinding performance as children become young adults.

We can only speculate about the commonality between the small-scale measures and the route learning task exhibited by our child participants. It is likely that small-scale spatial abilities directly contribute to wayfinding activities. For example, route learning requires individuals to perceive, remember, and manipulate spatial relations (e.g., Siegel and White, 1975), which are similar to processes measured by the small-scale spatial tasks. Hence, the association between measures may be relatively direct. However, it is also reasonable to think that the correlations reflect similarities in the developmental timing of improvements in the small- and large-scale spatial abilities. Many small-scale abilities undergo considerable development between the ages of 6- to 12-years-old (Newcombe et al., 2013). Large-scale spatial abilities such as route learning develop across the same age (see Allen et al., 1996; Kirasic, 2000). Given that both improve during the same developmental time frame, then correlations among abilities may reflect a common developmental process rather than a dependence of one set of abilities on another. For example, if both are closely related to improvements in more general cognitive abilities such as working memory that occur during the age range tested, then the correlations we observed may reflect a general developmental trend rather than any direct relationship between small- and large-scale spatial abilities. Purser et al. (2012) have also suggested that the correlations that they found may have been a product of individual differences in the central executive component of working memory common to the small-scale abilities that they measured and their wayfinding task. This relationship requires closer examination in the future.

### Relationship between Verbal Memory and Route Learning

We also observed that verbal memory accounted for a significant portion of the variance in route learning performance. Previous research with adults has suggested that route learning efficiency can be hampered by concurrent tasks that involve verbal memory. For example, Meilinger et al. (2008) found poorer route memory of a passively presented path if it was accompanied by a concurrent lexical decision task (see also Deyzac et al., 2006). Purser et al. (2012) have also reported a correlation between route learning and digit span for children 5–11 years of age. It is reasonable to presume that some aspects of route learning involve verbal memory to facilitate coding and maintaining the sequence of landmarks and turns that are encountered (Van Doorn and Blokland, 2014). In fact, having landmarks available and having landmarks verbally labeled benefits route learning of children and adults (Jansen-Osmann and Wiedenbauer, 2004a; Lingwood et al., 2015). However, it is also important to point out that the relation between verbal memory and route learning observed in the current study was only apparent for girls in our sample. This particular feature of the relationship is discussed in the next section.

### Sex Differences in Route Learning

One important difference between girls and boys observed in the present study was that boys performed better than girls in the route learning task in our virtual environment. This result is consistent with several previous studies (e.g., Gibbs and Wilson, 1999; Beilstein and Wilson, 2000; Choi and Silverman, 2003; Jansen-Osmann and Wiedenbauer, 2004a). Several other facets of our data are particularly interesting. Our results indicated that differences between girls and boys are evident at a relatively young age (6-years-old) and do not change significantly from 6- to 12-years-old. Our comparison of sex differences across the different age groups supports this conclusion. Hence, it is clear that any attempt to identify precursers of sex differences in wayfinding will need to target even younger children and look to biological predispositions or to experiential differences that are present by the age of 6 (Levine et al., 1999; Newhouse et al., 2007; Moore and Johnson, 2011). Further, the lack of change in the magnitude of the sex difference with age also suggests that normal developmental experiences of 6- to 12-year old children do very little to impact the sex difference we observed. This is not to say that the sex difference is immutable. Except for limited circumstances (e.g., scouting), wayfinding is not like academic skills that are purposefully instructed and rewarded when high levels of achievement are reached. Hence, unless an individual exhibits very poor wayfinding skills (i.e., is always getting lost), it seems unlikely that the way children approach wayfinding during this age range will undergo dramatic shifts. It also appears that the sex difference was not related to the small-scale abilities that we measured. Hence, unlike what is often observed for adults (e.g., Hegarty et al., 2006), the sex difference we found in wayfinding cannot be attributed to measureable differences in basic spatial abilities. Therefore, performance differences between girls and boys in the route learning task reflected differences in how boys and girls used their abilities to perform the task.

The results of the SEM analyses support the proposition that girls and boys approach wayfinding using different skills. We found that verbal memory had a stronger association with route learning for girls relative to boys and spatial ability had a stronger association with route learning for boys relative to girls. In addition, small-scale spatial ability and verbal memory equally contributed to route learning for girls. For boys only small-scale spatial ability contributed to route learning. This pattern of results is consistent with numerous adult studies of sex differences in wayfinding that indicate a general strategy difference in the way that males and females approach wayfinding activities. Men tend to engage spatial abilities to produce better configural representations of the environment whereas women tend to engage verbal strategies to encode information about landmarks and relative turns (e.g., Miller and Santoni, 1986; Ward et al., 1986; Galea and Kimura, 1993; Lawton, 1994, 1996; Brown et al., 1998; Dabbs et al., 1998; O’Laughlin and Brubaker, 1998; Prestopnik and Roskos-Ewoldsen, 2000; Ramos-Loyo and Sanchez-Loyo, 2011). It is probably not appropriate to attribute the difference we observed between girls and boys to overt strategic behavior, which generally implies a thought out and deliberate plan. Rather, it seems that this may be an early developing preference or propensity for visual information processing in boys relative to girls. There is certainly ample evidence to suggest that girls and boy engage different information processing tendencies to perform a variety of activities. For example, boys are more likely than are girls to rely upon visual information processing during reading (Huestegge et al., 2012). In contrast, girls exhibit a greater focus on details when drawing objects (Lange-Küttner and Ebersbach, 2013). These general tendencies in young children have the potential to transition to more deliberate strategies as children get older, resulting in the common sex differences observed in adult performance in skills such as wayfinding.

Does verbal memory actually interfere with route learning? It would seem that verbal memory contributes more to girls’ than boys’ performance and girls perform more poorly than do boys in route learning. Hence, this may be a reasonable speculation. However, it is also the case that girls who score higher in verbal memory perform better in the route learning task than those who score lower in verbal memory. Thus, engaging verbal memory does not directly lead to poor performance in route learning. It seems more likely that the girls’ poor performance has to do with the failure to engage sufficient spatial abilities in support of route learning. Why girls rely so heavily on verbal processes to perform route learning tasks and how verbal memory results in poorer performance levels are matters for future investigation. Perhaps a comparison of the route learning performance of girls and boys under conditions in which spatial and verbal processing are selectively disrupted would help to clarify these issues.

We suspect that these differences may apply to two aspects of real world wayfinding for girls and boys. First, it is reasonable to think that the use of spatial strategies will more readily translate to survey knowledge (Hegarty et al., 2006). Hence, boys would have a distinct advantage in developing an overall view of their environment. Survey knowledge is generally considered to be more flexible than route knowledge because it provides a means for developing alternative routes to different locations (Chrastil and Warren, 2011). In addition, boys would be more likely to develop a better overall “Sense of Direction” (Hegarty et al., 2002) and likely be more comfortable navigating the environment. Second, differences in one’s perception of their ability in wayfinding, often reflected in sense of direction, would likely lead to differences in a personal willingness to engage in wayfinding activities. It seems reasonable to conclude that boys would therefore be more likely to engage in a wider range of wayfinding activities and thereby develop relatively better skills as they get older. If the opposite is true for girls, then it may lead to anxiety about wayfinding, which is known to disrupt wayfinding performance in young women (e.g., Lawton, 1994, 2010). It may be worthwhile to evaluate when differences in wayfinding confidence emerge during the developmental period and how they translate into variations in wayfinding behavior.

## Limitations

Two general limitations are important to recognize. First, because sex differences were observed at the youngest age tested, it was not possible to identify precursors to the emergence of these wayfinding differences. Future research will need to apply procedures that can evaluate various aspects of environmental learning and wayfinding in very young children (e.g., Rieser et al., 1982; McKenzie and Bigelow, 1986) to determine at what age sex differences in wayfinding-related skills first become evident. Second, it is always necessary to be cautious about generalizing results from virtual environment tasks to real world wayfinding. There are many benefits to using a virtual environment to study wayfinding. However, it is not possible to capture the richness of the real environment with all of the variables that can both enhance and interfere with wayfinding. It will therefore be important to replicate the current results in procedures that more closely approximate real world wayfinding. In addition, it will be useful to consider the practical significance of these results to identify whether or not efforts to limit the sex differences through training would be of value.

## Conclusion

Whatever the base cause of differences in route learning between males and females as adults, we know that they appear at a very young age. Based on our data, significant differences are apparent at 6 years of age. Although these differences are likely to undergo some changes regarding how they are expressed, we observed possible antecedents of adult differences in the performance of children. Spatial ability predicted route learning better for boys than for girls and verbal memory predicted route learning ability better for girls than for boys. These differences may lay the foundation for the strategy differences in wayfinding typically observed between adult males and females. Adult males are likely to engage spatial-oriented strategies, and adult females are likely to engage non-spatial or verbal-oriented strategies when performing wayfinding tasks (e.g., Lawton, 1994, 2010; Dabbs et al., 1998; Bosco et al., 2004). An important question that remains and is worth exploring in future studies, is whether these differences can be modified and what type of intervention or exposure would be necessary to improve wayfinding performance of girls, especially at an early age.

## Author Contributions

EM contributed to design, testing, analysis, and write-up of the MS. YY contributed to design, testing, analysis, and write-up of the MS. BR contributed to design, analysis, and write up of manuscript. SS contributed to design and testing.

## Conflict of Interest Statement

The authors declare that the research was conducted in the absence of any commercial or financial relationships that could be construed as a potential conflict of interest.

## Footnotes

1 As recommended by Kline (2011, also see Hegarty et al., 2006), because there was only one indicator of the latent variable verbal memory, the error variance of the latent variable was fixed using the formula X = (1-r) (Sx^2^). In this formula, r is the reliability and Sx^2^ is the variance of the observed variable.
